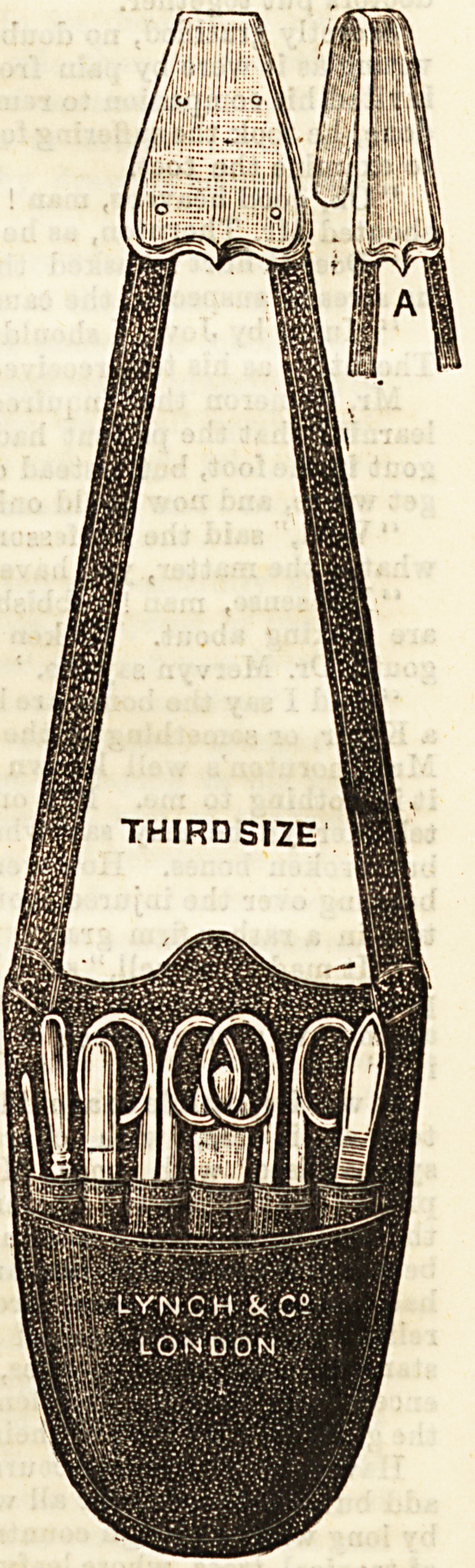# The Hospital Nursing Supplement

**Published:** 1893-04-08

**Authors:** 


"The Hospital" Nursing Mirror.

Being the Extra Nursing Supplement of "The Hospital" Newspaper.

[Contributions for this Supplement should be addressed to the Editor, The Hospital, 428, Strand, London, W.C., and should have the word "Nursing" plainly written in left-hand top corner of the envelope.]

### News from the Nursing World.

### A ROYAL INSPECTOR.

H.R.H. the Prince of Wales this week visited the workhouse at Dunmow, Essex. He thoroughly inspected every department, including the sick wards. The Prince carefully examined the dietary tables, clothing, bedding, &c. H.R.H. expressed great satisfaction at the appearance of the children and commended the humane arrangement of sending them to the elementary schools of the town without the uniform which is generally a conspicuous "badge of pauperism."

### ATTRIBUTES TO AIM AT.

Those who imagine that "taking up nursing" can be easily done at any time, and by almost any woman, may lay that delusion aside. Nursing is a serious and an all-engrossing profession, and the qualities requisite can be summed up in the description given by Dr. T. S. Billings regarding the demands made by the profession of medicine on students "for good mental capacity, for a long period of study, for much patience, for powers of physical endurance, for quick and keen sympathies, for honesty and purity of thought, word, and deed." These attributes are all needed for the ideal nurse, although for her a shorter period of study will suffice.

### HOW NOT TO NOURISH BABIES.

The nourishment which babies should get, and the food which they actually receive, are two very different things. We were told the other day of a baby of five weeks old who was "rather out of sorts." Naturally a question as to the diet was asked, sickness being one of the symptoms. "Well, mum," said the mother, "he haven't had anything harmful. He seemed but poorly yesterday, and so his father walked eight miles to A—— and bought a nice piece o' plaice, and we biled it and beat it up and give it to the baby. That's all he's had. Last week we give him some rabbit's brains beat up fine, and he kept them down. He hasn't bin quite the thing since, so I thought a little fish might suit him better." "But why not keep the baby to milk?" "Bless ye, mum, babies like a change. I allers gives mine a bit o' all sorts." Poor baby, and unhappy mother! We rejoice whenever we hear of an intelligent nurse giving practical instruction in the art of baby feeding.

### A GOOD BEGINNING.

The Nursing Association at Berwick-on-Tweed has done a successful first year's work, and there is every reason to believe that it will soon largely increase the field of its labours, for it is managed most ably. The fact that Boards of Guardians are now empowered to keep a nurse for outside work was brought forward at the meeting. With the help which can reasonably be demanded from the Workhouse Governors, and also with the co-operation of the Infirmary authorities, whose out-patients benefit immensely by the Nursing Association, there should be no difficulty in securing a second Queen's nurse, and she is urgently needed at Berwick.

### PROGRESS OPPOSED.

The nursing arrangements at the Bath Workhouse have been justly criticised by the medical officer, as we said in our issue of March 18th, under the heading "Difficult Nursing." But the troubles are no nearer removal than they were then. One of the committee protested at the last meeting against having it "all stirred up again. He had been a long time a member of the board, and he never knew any thing of the kind." We venture to think the confession no credit to the board, and the sooner a little more "stirring up" takes place the better for the paupers.

### NOVEL TRAINING SCHOOLS.

"How do you define the word training?" is a query which would receive a startling reply if given by members of some Boards of Guardians. At Totnes, as we mentioned the other day, the wash-tub was considered a test for nursing capacity. Bath is not going to be outdone by Totnes in fertility of resource, as the following extract shows. It is taken from the *Bath and Weekly News*, which briefly sets forth the unsatisfactory position of medical officer, master, and Board, concluding: "I find that there are only two nurses to look after nearly two hundred sick and invalid patients, and one of these is a young girl of about 20, who only a few months since, I am given to understand, was practically taken out of a milk-cart to perform, without any training, the duties of a nurse." At the Farm Colony, organised by General Booth, the hospital containing 18 beds is in the charge of an untrained woman whose experience has been gained by "being about a great deal and picking up things here and there."

### WHAT WILL THEY COST?

This question was asked a good many times in the course of a recent meeting of the Portsea Guardians. The advantage of instituting trained nurses for sick paupers was under discussion. The poor, as well as the rich, should equally enjoy the advantages of skilled attendance. Some of the Portsea Guardians realise this, and we wish them success in their benevolent attempt to introduce qualified nurses. But they must not rest content with the terms of the initial proposals. They certainly should not let the statement that one nurse is sufficient for eighty or one hundred patients go unchallenged, and they ought not to expect applications from any duly qualified persons for posts at £20 per annum. There is a general outcry at present for more infirmary nurses. The supply of suitable women will never meet the demand until Guardians have learnt that adequate pay must be given to skilled workers, and also that the individual responsibilities of each nurse must be reduced within reasonable limits.

### HOW TO SPREAD INFECTION.

There are many effectual methods of attaining this undesirable end, but few are more ingenious than the simple one selected at Leicester. This consisted in allowing certain officials connected with the Infectious Hospital to sleep at their own houses in the town. At the same time a system of quarantine for other persons was in force. This reveals a fine inconsistency probably somewhat unique in these days of sanitary enlightenment—in England, at any rate. Until the present small-pox epidemic necessitated the erection of a "Humphrey's" building for the nurses, these attendants fed and slept in the same building with the patients suffering from this dreadful disease. "Dreadful," according to modern science, and "preventable" too, but intelligent Leicester apparently neither dreads nor prevents the visitation of this scourge of mankind.

### WOMAN'S SHARE.

The words, "a Nursing Guild," sound pleasant in our ears, and in reading them we hope to peruse some practical schemes. The plan sketched out in the recent lecture at Redruth will need many modifications if it is to meet the needs of the present day. When trained nurses were scarce, and Mrs. Gamp reigned, most of the skilled attention received by the sick came direct from the doctor's hands, consequently general practitioners of those old days were framed by experience (often dearly bought) into very good all-round nurses. This has changed now, the professional man confines himself to his own share of the work, and leaves the woman to do her portion, having proved that the intelligent, well-trained nurse knows how to carry out his directions, and her poultices and bed-making are superior to his. We would, therefore, venture to advise the people at Redruth, if they wish for a course of nursing lectures which shall help untrained ladies to be of service to their neighbours, to secure a thoroughly qualified and experienced nurse to show them how best to carry out this essentially womanly and most important work. There are many details which can only be adequately explained by a woman speaking to women. These must of necessity be omitted in the popular nursing lecture, which is all that can be attempted by teachers of the opposite sex.

### VOLUNTARY SUPPORT.

The Nursing Association at Kirriemuir is growing very popular, and unsolicited subscriptions and donations have been cheerfully offered to this admirable society. The demand for Queen's nurses has been so great during the year that Kirriemuir only succeeded in getting one for seven months. The record of work done in that time is excellent.

### AN UNFULFILLED PROMISE.

The nurses at Belvidere Fever Hospital have aired what seems to be a genuine grievance in the *North British Daily Mail*. The general public were informed through the newspapers, in the course of last autumn, that a higher rate of pay would be given from January 1st to secure "experienced certificated nurses" to remain longer on the staff. But the increase has not been made. "The nurses are still paid at the old rate, and they have been unable to obtain any reason or explanation why the promise has not been fulfilled." If the same nurses have remained at the hospital, there cannot possibly be "any reason" for which a cheque for the balance due to each worker cannot prove the best "explanation."

### NURSES IN WALES.

The Welsh branch of the Q. V. J. I. has just completed the training of four probationers, one of whom speaks Welsh. Two have received Queen's badges. The annual meeting at Cardiff had to consider the question of the funds, which seriously require augmentation. Of the work already accomplished by the Association warm appreciation was universally evinced, and Mr. John Cory, J.P., who presided on the occasion, pointed out that there was no "red tape" or ceremony about district nursing. When a sick person needed a nurse a note sent to the Home secured prompt and skilled attendance for the poor sufferer, "poverty and sickness being the only recommendations needed."

### ECONOMY AT MELBOURNE.

The financial outlook at the Melbourne Hospital is somewhat gloomy just now, and various reductions are suggested in the expenditure. A proposal to decrease the nursing staff and the salaries has not yet received consideration, and we hope this may not become necessary. The small wards, which are attached to the large ones, are to be reserved in future for patients who can pay at the rate of thirty shillings a week. Certainly there must be many sick persons in Melbourne whose friends would gladly meet this modest charge for skilled treatment and care.

### GOOD NEWS FOR KANDY.

A most excellent step has been taken by the hospital authorities at Kandy, Ceylon, in arranging to place the entire nursing in the hands of fully trained and qualified women. A party of East Grinstead Sisters has accepted the charge, and will take out only thoroughly trained nurses. After the extraordinary arrangement made at Colombo, for an untrained Sisterhood to look after the Government Hospital, it is encouraging to hear that beautiful Kandy is to have better luck.

### NURSES AT BERLIN.

The number of nursing Sisters at the Victoriahaus, Berlin, has been increased to 160; of these 94 are employed in Berlin hospitals, whilst the others work in institutions elsewhere. The interest of the Empress Frederick in this association is well known. Nurses were sent from the Victoriahaus to Hamburg during the cholera, forming another instance of the truth of our frequent assertions that application for aid in epidemics will assuredly never be made in vain to existing institutions.

### "THE HOSPITAL" ENDOWED BED.

We beg to remind our readers that subscriptions to the Nurses' Bed are now due. Contributions sent to the Editor, 428, Strand, will be acknowledged in The Hospital. £1 received from Nurse Catherine, making total up to date £14 18s.

### Management of Consumption.

### IX.—THE FEEDING OF CONSUMPTIVES.

In olden time, before the true nature of consumption was at all understood, it was the universal practice to deplete the patient by various means, such as bleeding and starvation. It was thought that because a man with consumption was rapidly wasting, and had high hectic temperature, that was sufficient reason for exhausting him in every conceivable way so as to quench the raging fever within him. Now all that has changed, and if one idea is more fixed than another it is this, that a consumptive patient must be tempted and encouraged to eat an abundance of nourishing food, and that so long as this food can be assimilated, the patient may be said to be maintaining his ground. Our object in this paper is to point out the rules which should guide us in feeding consumptive people, and the kind of food they should eat.

For practical purposes of dietetics consumptives may be divided up into three great classes: 1. Consumptives who have little or no gastric disturbance, and no complications. 2. Those having much gastric derangement, and complication. 3. Advanced consumptives. A consumptive patient almost always complains of symptoms pointing to stomach disorder; he complains of pain after food, constipation, a feeling of sickness, amounting sometimes to actual vomiting. His tongue is almost always furred, and his teeth are in bad condition. A fair proportion of them have tubercular ulceration of the bowels, and as a consequence, diarrhœa, which often proves most intractable. In addition to this, phthisis itself is almost always attended with great aversion to certain kinds of food, and loss of appetite generally.

The consumptive is thus handicapped; he is the victim of a disease in which it is absolutely necessary that he should assimilate his food in order to make headway against his complaint, and yet that very disease produces a condition of his stomach which prevents him digesting his food successfully.

From this one sees at once the importance of knowing what food is most likely to prove suitable, and how such food should be given.

For the first class, those having little dyspepsia and no complications, a liberal diet may be allowed.

A scheme of dietary upon which they should not fail to improve would be somewhat as follows:—

Breakfast: Cocoa or coffee, porridge, bacon, bread and butter, and an egg lightly boiled. At eleven o'clock, half a pint of milk. At one or two o'clock the patient should take his lunch or dinner, at which he may have lightly-cooked mutton and boiled fish. He should eat sparingly of potatoes, and for drink, may be allowed half a pint of bitter beer, or, if his means permit, a glass or two of claret or hock. Beef is very nearly certain to disagree with him, and all such things as pastry should be avoided. He may have custards and jellies, and may eat, in moderation, stewed fruit or grapes. In the afternoon he may have a cup of tea, with toast or bread and butter. In the evening, whether it be dinner or supper, his rule should be to take such plain and wholesome food as has been recommended for dinner. The last thing at night he may have another half a pint of milk or beef-tea. He may take cream and fats at all times if he can fancy them.

If, on the other hand, the patient has had dyspepsia, or is suffering from some of the complications, such as high temperature or diarrhœa, then all the arts of persuasion the nurse has at command will be required to coax him to feed. Let us take the case of a consumptive whose stomach rejects everything it receives. Sometimes the sickness is only slight, and may be effectually prevented by getting the patient to lie in a recumbent position for a while after each meal. In other cases nothing short of systematic feeding will have any effect. The patient is going down hill from want of food, and unless it can be rectified, will proceed from bad to worse.

When incessant vomiting comes on, all idea of taking regular meals must be abandoned. The principal articles of diet must be milk and meat essence. The quantity to be consumed in the day should be about three pints of milk and a pint of meat essence. The patient, of course, must be in bed. To the milk may be added a third of soda water, lime water, or barley water. The correct plan of feeding consists in giving a small quantity of milk and beef-tea at frequent intervals. One may begin with three ounces of milk and an ounce of beef tea, or other meat essence, every hour. So long as the patient can retain this without feeling sick, all is well, and the quantity may be gradually increased until five ounces of milk and two ounces of beef-tea are taken every hour. The moment sickness comes on the quantity must be decreased until, by constant varying, the exact amount is known which, when taken, will not cause sickness. Sometimes even small quantities of beef-tea brings on sickness; in that case one may try the various meat extracts, all more or less efficient, or sometimes every kind of meat essence has to be dispensed with and milk alone administered. It will be found very useful sometimes to boil the milk twice before giving it; in this way the germs causing the milk to become sour are killed, and it is much better borne by an irritable stomach. It must be remembered that however useful drugs are in the treatment of sickness, they are nevertheless inoperative unless the patient is at the same time fed in a proper manner. As the stomach becomes stronger more and more may be given, and finally the patient is able to take custard, jelly, toast, and fish, or he may be allowed to chew lightly-cooked beefsteak, but not swallow it. In this way his powers of digestion rapidly improve, and before long he is able to take ordinary wholesome food, and the result is a gain in weight in the majority of cases. If the appetite of a consumptive is to be kept in good order, it is requisite for him to take healthful outdoor exercise, and to smoke in moderation. The consumption of large quantities of tea cannot have anything but a baneful effect upon his stomach. Milk is always of use to the patient, and not less than a pint and a half should be taken in a day. If he can take them he may have cream and grapes. This latter fruit has gained just celebrity in the treatment of consumption, and is spoken of as "the grape cure." It is said that patients seldom fail to put on weight when taking large quantities of them.

Coming now to the feeding of a patient with advanced consumption, all our efforts are of no avail; it is impossible to contend successfully with a total loss of appetite, where the most tempting morsels are viewed with disgust. Yet a nurse would be sadly lacking in duty did she not bestow just as much pains upon this hopeless case as on one who will repay her for her trouble. Here there is not much object in choosing a suitable diet for gaining flesh, and almost anything which will tempt the patient may be given. An excellent drink for the consumptive is Koumiss; this fermented milk of mares allowed to run wild on the Kerghis steppes of Russia. A good copy of it is the following: Take good cows' milk, nearly fill a strong bottle with it, add a spoonful of lump sugar and a piece of yeast about the size of a bean, shake well and cork down, shake twice a day for six days. The Koumiss should be ready for drinking on the sixth day. It has been claimed for this beverage that it has a curative action upon consumption. We have hitherto said nothing about alcohol, but it is very necessary in cases of weakness and prostration to administer an ounce or two of brandy or whisky in the day. Rum and milk is another good stimulant. Port wine may also be taken in moderation, and sound bitter ale or stout. It very seldom happens that a case of consumption requires more than five ounces of spirit a day, and the greater number improve on only two. To recapitulate, then, it is important to feed consumptives carefully, with sound wholesome food if they can bear it, with light food if their stomachs are irritable. By doing this the nurse will frequently have the pleasure of seeing her patient gain in weight.

### Jottings from the Punjab.

Perhaps some of the readers of The Hospital may be interested to hear of the work done by the Sisters belonging to the Lady Robert's Nursing Staff, who during the hot season are stationed at Murree. Here an officers' hospital for the reception of sick officers requiring skilled nursing and home comforts is established. The hospital is under the supervision of the principal medical officer in charge of the station, and the household arrangements are under the management of a Lady Superintendent. Two Nursing Sisters are attached to the hospital, who undertake the whole of the nursing, with the occasional assistance of soldier orderlies. Of the 40 patients admitted, most of them were enteric fever cases, the others accidents. For instance, one was an officer who was accidentally shot in the leg while attending a musketry class, and so severely injured that his leg had to be amputated, and he was afterwards nursed in the hospital. Another was the case of a young subaltern who, while riding along one of the Murree roads, was thrown off his pony and fell down the mountain side, his pony falling on the top of him. He was immediately taken to the hospital, where it was found that, besides being severely cut and bruised, he had fractured one arm and dislocated the other. All the officers testify to the kind attention received from the nurses, and say how well their comfort is looked after. No infectious or cholera cases are admitted, but during the cholera epidemic of last summer one of the Nursing Sisters was sent to assist the then overworked army doctor with some of his cases. Her services were greatly appreciated by him, as well as by the patients and their friends. Only those who have themselves experienced an epidemic of cholera can have any idea what a strain the nursing of these cases becomes. The work is most arduous and trying. Of these nurses we in India say:

> "Honour to those whose words and deeds
> Thus help us in our greatest needs."

### Where to Go.

Free Lectures.—Dr. Symes Thompson will lecture on "Physic," at Gresham College, E.C., at six p.m., on April 11th, 12th, 13th, and 14th.

"Niobe" at the Strand Theatre is an entertainment calculated to drive away every memory of work and worry. To a tired nurse this charming play will bring refreshing mirth. The fun is irresistible, and the wit is clean and wholesome, and there is plenty of it. The short first piece, "No Credit," is also quite a treat in its way, and we commend the Strand Theatre to our readers' notice.

Lecture at Trained Nurses' Club, 18, Buckingham Street, Strand, on April 21st, at 7.45 on "Diseases and Management of New Born Infants." By Dr. Robert Lee. Tickets for non-members, 6d.

In connection with the forthcoming dinner to be held at the Holborn Restaurant on Tuesday, May 9th next, in aid of the North-Eastern Hospital for Children, it has been decided to ask ladies to co-operate as stewards. The Lord Mayor, who will preside, will be accompanied by the Lady Mayoress.

### Death in our Ranks.

Nurse Jessie Parkinson, aged 28, died on Good Friday at St. Mary's Nurses' Home, Plaistow, E., of scarlet fever, caught while discharging her duties, after only a few days' illness.

### Nursing at Natal.

### II.—A PRIVATE NURSE'S OPINION.

There was also a well-worn but rather dangerous-looking path winding down the side of the gorge; though I need hardly say that at the time neither of us felt equal to its descent, though Eric, whose spirits were rapidly rising, sorely wanted to make the attempt, but his anxious mother kept him securely placed between us, and never relaxed her hold upon his hand. Far below us the river, already forgetful of its recent passion, flowed placidly along, looking in the sunshine like a broad band of silver.

Owing to the formation of the ground over which it rushes, the waterfall is not quite uniform in its descent—a much larger body of water falling on one side than on the other, the consequence being that one-half of the stream is dark and turbid, while on the other side it is bright, clear, and sparkling. We amused ourselves by drawing comparisons from this circumstance.

Mrs. Clarke, who is inclined to be romantic, said she was reminded of the different qualities of the sexes—of man's strong, earnest purpose, and deep, if sometimes turbid, passions, in contrast to the lighter and more graceful gifts of womanhood. I thought that the waterfall symbolised human life with its hours of tempest and rough trial, interspersed with moments of bright sunshine and exquisite happiness.

And then the river below, winding so peacefully along, reminded me of the inner Christian life, uninjured by passing storms, flowing onward "like a silver-clear stream into the solemn sounding main, into the ocean of eternity." But Eric, who is nothing if not quaint and funny, brought us back from these flights of imagination by saying in his most prosaic tones that the waterfall reminded him of the water in his bath after he had been making mud houses—so bright and pretty when Jantje poured it in, and so black when it came out again! After this we could not sentimentalise any more, but sat for some time longer enjoying the fair scenery and the fresh, invigorating breezes. For many miles around the open country lay before us in the clear atmosphere, dotted here and there by cosy-looking homesteads, which peeped through plantations of gum and wattle trees, and a little way up the river were the white tents of the regimental quarters.

All was bright, calm, and beautiful, and none but those who have known what it is to pass weeks in a house of sickness and fear can, I think, understand how keenly we enjoyed that first morning of emancipation and rest.

We remained out till past noon, for the unusual freshness of the air in the neighbourhood of the Falls enabled us to do this with comfort, and returning to our hotel, again enjoyed a hearty meal, and spent the afternoon resting in a quiet and cool sitting-room, which had been placed at our disposal. I must not close this description of our first day at Howick without mentioning an amusing story which enlivened our dinner table at night.

Having seen our little convalescent to bed and fast asleep, Mrs. Clarke decided that we should dine at the table d'hôte. Here we found our fellow traveller by the train, Professor Cameron, who was an old acquaintance of Mrs. Clarke's. Mr. Cameron had been further up the line on business, and having just returned to Howick, intended sleeping there and proceeding to Maritzburg by the early morning train. After some desultory conversation he told us, in his peculiarly dry, quaint manner, what a splendid chance he had that day met with of "scoring one against his ancient enemy," as he called Mr. Thornton.

It seemed that while we were exchanging to the omnibus at Howick Station he had alighted for a few moments' walk up and down the platform. Seeing him pass and repass, Mr.

Thornton had, with evident embarrassment, accosted him thus:—

"Oh! by the way, Cameron, I wish you would change your seat to this carriage if you are going on. I'll be glad of a few minutes' conversation with you."

Supposing there was to be a renewal of some old political skirmish, and nowise averse to the fight, if it were forced upon him, the professor acquiesced. But no sooner had the train resumed its course than Mr. Thornton, whose lameness we had all noticed at the City station, lifted up a swollen and bandaged foot, saying,

"The fact is, I want you to look at my foot. I've been suffering horribly with it. Gout, Mervyn says, but it does not get better, and I'm not satisfied. I wish you'd have a look at it. I believe you know more, after all, than all the doctors put together."

Secretly gratified, no doubt, by this tribute to his intellect, wrung as it were by pain from his "enemy," Mr. Cameron invited his companion to remove his bandages. This being done, he took the suffering foot between his hands, and began to examine the toes.

"Oh, good heavens, man! take care what you are about!" shouted Mr. Thornton, as he winced with agony.

"Does it hurt?" asked the Professor innocently, though he already suspected the cause of suffering.

"Hurt? by Jove, I should think so indeed!" groaned Mr. Thornton, as his toes received another gentle pressure.

Mr. Cameron then inquired into the history of the case, learning that the patient had been treated for some time for gout in the foot, but instead of finding relief seemed rather to get worse, and now could only limp about with difficulty.

"Well," said the Professor, "if you want me to tell you what is the matter, you have broken two of the bones."

"Nonsense, man! rubbish! You don't know what you are talking about. Broken bones, indeed! I tell you it's gout; Dr. Mervyn says so."

"And I say the bones are broken. You have been kicking a Kaffir, or something of the sort." (This was a sly hit at Mr. Thornton's well known irascibility of temper.) "But it is nothing to me. Put on your bandages, go home, and tell Mervyn he may say what he likes. I say it is not gout, but broken bones. However, let me look once more. And bending over the injured foot, Mr. Cameron again seized the toes in a rather firm grasp.

"It made him yell," said he to us, "but"—with an expression of inimitable drollery—"I thought I should never again have such a chance of paying off old scores, and I took it!"

It was impossible to avoid laughing heartily over this story, told, as it was, with characteristic raciness, though one sympathised with poor Mr. Thornton. But the result proved that Professor Cameron, who though not practising the healing art, was a most accomplished scientific man, had been correct in his opinion, and it was not until the patient had been treated for "broken bones" that he obtained relief, and the free use of his walking powers. I understand that the two "enemies," having since settled old differences, are now on excellent terms, working together for the good of "the land of their adoption."

Having described the course of one day at Howick, I need add but little more, for all were much alike, though varied by long walks through country lanes, overshadowed by rows of tropical trees, whose leafy branches cast refreshing shade upon the road. Then there were visits from and to the kindly residents of the neighbourhood, with most of whom Mrs. Clarke, who had spent nearly all her life in Natal, was acquainted, and there were tennis and polo matches continually taking place between the civilians and the military men then stationed in the village.

We often revisited the waterfall, being never weary of its beauties, and we soon learned to descend the narrow, precipitous path to the bottom without any fear of giddiness. We lingered on until the rosy hue of health became confirmed upon our little patient's cheeks, and when we left it was with a new store of strength, both physical and mental, to meet the duties and emergencies of the future. One incident only occurred during our stay to sadden us; it was when the body of a missing soldier was found lifeless and battered at the foot of the Falls. No one could tell what had lured him to destruction. Whether he had wandered at night too near the fatal edge, or whether, for some cause "weary of living, so weary," he had voluntarily overstepped the margin, none would ever know. At any rate, he received the benefit of the doubt, and was buried on the following day with military honours. For a few hours this event cast a gloom over the little village. One could not help speculating about the unfortunate man—his individual life, perhaps unknown to the closest comrade; the friends at home, mother, wife, or sister, to whom his death would be an agonizing blow. But life goes on with startling rapidity, and long before we left the place the circumstance, if not quite forgotten, had ceased to occupy public interest.

We bade adieu to Howick with real regret, and sincere wishes for a renewal some day of our pleasant holiday there. On our return journey from the hotel we happened to be the only occupants of the omnibus that had originally conveyed us thither, but before we had proceeded half way to the station we came upon the "Castle" 'bus—still unfortunate, though it had secured half-a-dozen fares, for its "disselboom" was broken, and the passengers were sitting disconsolately by the roadside, waiting for assistance. We took them and the mails on with us, leaving the rival vehicle behind, a melancholy spectacle which moved our driver to pity, and silenced his customary "chaff."

Such a holiday as I have described is, of course, as rare as it is delightful to nurses in Natal, as elsewhere; but while I enjoyed it I thought often of my sister nurses in London hospitals, wearied and over-strained, as I knew they must often be (for I, too, had worked amongst them), and I wished they could share with me this free, untrammelled life, and pure, sweet air. A country holiday in Natal is no doubt wanting in variety and excitement, but to all who recognise in Nature the best healer and consoler, such a holiday as I have tried to describe must be of priceless value.

### Notes and Queries.

### Queries.

(96) *Dispensing.*—Will you kindly tell me what qualifications a lady dispenser requires, and if there is more than one she can take?—*W. J. B.*

(97) *District Nurse.*—Are district nurses supposed to supply and pay for their *locum tenens* during their holidays?—*K. G.*

(98) *Chicago.*—I see in The Hospital of March 25th that there are to be trained nurses taken on for the hospital in connection with the Chicago Exhibition. I should be much obliged for any information.—*Mrs. E. P.*

(99) *Nursing.*—A book wanted for study by a would-be nurse.—*Novice.*

(100) *Certificate.*—Will experience in a sanatorium count as training?—*M. S.*

(101) *Mental Attendant.*—I want training for this work, and shall be glad of any information as to this, and as to how to get a post as useful lady companion.—*J. E. A.*

(102) *Photograph.*—I should be glad of advice as to best course to pursue to recover a photograph sent with stamped envelope for its return to a matron of a convalescent home. I applied for post of nurse, and cannot get back the likeness, although I have written again about it.—*Lady.*

(103) *Legal Notice.*—Is it under ordinary circumstances legal to receive a month's notice if salary is paid quarterly? Can you inform me?—*Kate.*

### Answers.

(96) *Dispensing* (*W. J. B.*).—The information you require is given in "Burdett's Hospital Annual," published by The Scientific Press, 428, Strand, price 5s. There was an article headed "How to Become a Dispenser" in The Hospital, September 24th, 1892.

(97) *District Nurse* (*K. G.*).—If you take your holiday at the slackest time of the year perhaps no substitute is needed, but should the association for which you work desire a "locum," doubtless they will select and remunerate one. It is always better to have a clear understanding as to holidays when taking an engagement. It saves much friction later on.

(98) *Chicago* (*Mrs. E. P.*).—If you read our paragraph carefully you will see that "taken on" is not the expression we use. There are plenty of trained nurses already in America. English representatives of nursing are cordially invited to the Congress, but there are plenty of Americans to take the ambulance department, and we have seen no official request for additional help.

(99) *Nursing* (*Novice*).—You had better get a good book on general nursing first, such as "Lückes' Lectures on Nursing" or Lewis's "Theory and Practice of Nursing," and afterwards Cullingworth's small "Manual for Midwives."

(100) *Certificate* (M.S.).—No. You must have a full training in a recognized hospital. Get "How to Become a Nurse," price 2s. 6d., at the Scientific Press, 428, Strand, London.

(101) *Mental Attendant* (*J.E.A.*).—You could get three months' training in theoretical and practical mental nursing at Berrywood Asylum, Northampton. Write to the Medical Superintendent for terms. When you are qualified you would do well to advertise in The Hospital.

(102) *Photograph* (*Lady*).—We should advise you to write again to the matron as clearly as you have to us. Describe your photograph, it may be mislaid amongst others.

(103) *Legal Notice.*—If an employé is engaged by the month, and the engagement is arranged to terminate by a month's notice, that would settle the question. It is customary to give a quarter's notice where the salary is paid quarterly in all other cases.

### Test Questions

GIVEN TO NURSES OF THE STATE HOSPITAL, UTICA, NEW YORK, PRIOR TO THEIR EXAMINATION.

Introductory Lecture.

1. What are the general qualifications of a good attendant?
2. What is "tact" and "self-control"?
3. What are the most important things you should learn in regard to obeying orders?
4. What are the duties of a charge attendant in regard to note-making in the ward books?
5. How should attendants conduct themselves when the medical officers make their rounds?
6. What can you say about the care of clothes' rooms?
7. How should you care for bedridden patients?
8. What are the rules in regard to going out with walking parties in winter, and about visiting from ward to ward?
9. How should you receive instructions and directions from the supervisors?
10. How should you treat patients at all times?

Anatomy and Physiology.—The Bones, Joints, and Muscles.

11. What are the bones composed of chiefly?
12. At what time of life and in what kind of insanity are the bones thought to be quite brittle?
13. Name two uses of the skeleton.
14. What arrangement has the spinal column which prevents jarring of the brain when one moves about?
15. Why should you be careful that too much pressure is not brought to bear on the chest of a struggling patient, by the knee or against an obstacle?
16. Name two things which make up a joint.
17. Mention as many reasons as you can why demented or other patients should not be allowed to retain the same position continually.
18. What uses do the muscles have?
19. How does exercise improve the general health, and where is it best taken?
20. What precautions must be used in regard to the occupations and amusements of patients as regards health?

Circulation of the Blood.

21. Name three of the digestive organs.
22. Why should food be finely chewed?
23. Why should food be slowly chewed?
24. What kinds of food, in what quantities, and how often should food be administered to the sick?
25. After our food has been digested, how does it reach the different portions of the body to nourish it?
26. What causes the blood to circulate throughout the body?
27. What is the difference between the blood in the veins and the blood in the arteries, and where does the venous blood become purified?
28. What direction does the blood flow in reference to the heart, in the arteries and in the veins, and how can you tell the difference between bleeding from a vein and bleeding from an artery?
29. What is respiration, and what organs are used in breathing?
30. Why is air that has once been breathed poisonous, and why is pure air necessary to health?

The Brain and Nerves.

31. What organs comprise the nervous system?
32. How is the brain protected from injury?
33. What portion of the brain as a whole is the active part? How is this portion increased in extent?
34. Name three things which are necessary to a healthy action of the brain?
35. What are the three principal parts of the brain called?
36. How is the brain nourished, and when does it obtain rest?
37. Name what are ordinarily called the "five senses," and tell what organ or organs preside over each?
38. Mention two uses which nerves have?
39. In what kinds of insanity is there less sensitiveness of the skin than is natural?
40. Mention three diseases of the nervous system beside insanity, which are found in the wards?

*(To be continued.)*

### Hints for Nurses.

The "Nurse's Companion" is the name given by Messrs. Lynch, 192, Aldersgate Street, to the strong case of sewn Morocco leather which is shown in our illustration. It is of very convenient size, and even when completely fitted up, is by no means unduly heavy. A nurse can attach it to her dress band, or to a leather belt, with every confidence that the deep nickel-plated hook at the top of the straps can be trusted to bear its weight. These cases can be bought without fittings to suit the purchaser who has already her own instruments. The one we give a sketch of here contains a wisely-chosen collection of well-finished and useful articles, namely, a probe, a director and ear scoop, a clinical thermometer in metal case, whose accuracy is guaranteed, a pair of scissors, a tongue spatula, and two pairs of forceps. The case itself is 4s. 6d., with straps complete, and with the contents we have enumerated it is sold at the moderate price of 18s. 6d., and we recommend it to the consideration of our readers as a particularly convenient "Nurse's Companion."

### Minor Appointments.

Port Said British Hospital.—Miss C. A. Millar has been appointed Sister at this hospital. Miss Millar was trained at the London Hospital, and has recently taken duty at the Mena House Hotel, Cairo.

Hull and East Riding Reckitt Convalescent Home.—Miss Margaret Alice Laycock has been appointed Matron of this Home. Miss Laycock was trained at the Royal Southern Hospital, Liverpool, and afterwards held the post of Matron at the Ellesmere Port Hospital, Manchester Ship Canal, and she was subsequently Matron at the Cottage Hospital, Bromsgrove. We wish Miss Laycock success in her new post, which her past experience well fits her to fill.

### Everybody's Opinion.

*[Correspondence on all subjects is invited, but we cannot in any way be responsible for the opinions expressed by our correspondents. No communications can be entertained if the name and address of the correspondent is not given, or unless one side of the paper only be written on.]*

### INDIA.

"A LIVERPOOL NURSE" writes: I should be very glad of any information respecting nursing homes and institutions in India from nurses with practical experience on the subject. I do not know to whom to apply for forms of application, or how best to obtain an appointment.

### CO-OPERATION.

NURSE OLIVE writes: In reference to co-operation of nurses in Birmingham, Nurse Elita writes she would like to discuss the subject, and I know of several nurses in favour of co-operation. I have had a talk with one of the leading surgeons in this city who heartily approves of the plan, and he would give us his support in the undertaking. I shall be pleased to discuss the matter with Nurse Elita; my address is—Nurse Olive, 8, Duchess Road, Birmingham.

### MANAGEMENT OF CONSUMPTION—EXPLANATION.

"DR. A. C." writes: With reference to the subject of linen for night garments, I beg to say that I did not mean to write the word linen; what I intended to convey was that the night-dress should be of calico, and that the sheets should be of that thick and somewhat rough variety generally known, I believe, as twill. Had I intended the word linen, the criticisms would have been much to the point. I do not object to flannel, provided it be of the thinnest description.

### HINTS FROM AMERICA.

"A NURSE IN CALIFORNIA" writes: It is indeed wise to get all the information possible before leaving England for America. I have been here two years, and can truly say that love for the pay and not for the nursing work is the ruling spirit of the Western States. I have been in Chicago, and intend going to the World's Fair if I can afford it. People in Chicago say that all the rates during Fair time will be very high. The cheapest way of living will be to find a room on arrival, or, better still, to write to the Bureau of Public Comfort, Chicago, at once for printed form. By paying a small fee, a furnished room will be assigned, for which the charge will be one dollar (4s.) a day. Meals can be had at a restaurant, costing from 25 cents. upwards. Breakfast and supper could possibly be cooked in your own room. A more expensive, though simpler, way of living is the boarding house; the charge is from eight dollars a week upwards. This includes a furnished room and three meals a day. A dollar is 100 cents, but single cents are seldom seen. The smallest money in circulation is the nickel five-cent piece. As to clothing, the most suitable is a costume that looks well in wet weather or dry, and a becoming hat to shade the eyes. Nursing costumes are often ridiculed. A bag with needful clothing only (that is, for a person sure of returning) will prove most convenient. Jackson Park, the scene of the World's Fair, is about three miles out of the city at the extreme south end. Including car fares, entrance fees, and other extras a nurse might manage fairly well on two dollars (8s.) a day. It takes twenty-four hours by rail from New York to Chicago. A spirit lamp, good English tea and sandwiches, or biscuits, will be useful.

### THE YOUNG NURSE.

"SISTER McT." writes: I read with great interest the "Young Nurse's" letter on herself. I am surprised at any well-regulated hospital allowing a nurse to enter at the age of seventeen, however "favoured" she might be. I was trained at a general infirmary where the nurses entered at twenty-five. Judging from personal observations there during nearly four years, I should say no nurse ought to begin before twenty-three. It is impossible to form any idea of the responsibility that may devolve upon them at any time. As for giving any nurse of nineteen full charge of sixty-eight or seventy-two patients, the bare idea is absurd; it is neither fair to the patients nor right for the nurse herself; the care needed by the one, and the rest necessary for the other, seems equally disregarded. When Nurse M. grows older she will be better able to appreciate the value of trite compliments from patients or commonplace platitudes from "shrewd Scotch doctors." We may then hope that she will refrain from quoting them. "Young, though not thoughtless," and "the life of the wards," and so forth, may be pretty autobiography, but I have heard it called by another name—at least, it is certainly not argument.

### For Reading to the Sick.

### RESURGAM.

IT was the custom some forty or fifty years ago, when a nobleman or other great person died, to put up his coat of arms, painted on a large black board upon the front of his house, with the word "Resurgam" underneath, which means "I shall rise again." It became in time a mere form and died out in consequence; but at first it must have given great comfort and consolation to the survivors, as every time it caught the eye they were reminded that he who had gone before was resting in the sure and certain hope of a glorious resurrection.

If we are Christians, we may see a bright and beautiful lesson for ourselves in this old custom, for as we are all the sons and daughters of the great King, we can use our Father's arms and the motto, "I shall rise again," since He has promised by His beloved Son that "Whosoever believeth in Me, though he were dead, yet shall he live."

The words have many cheerful interpretations for our bodies as well as our minds. For instance, when we are on a sick-bed we cling to them with a hope which helps on the cure, and we rise from what has been counted to be our death-bed and go forth with new life and vigour to our daily tasks. But this fresh life, so welcome, so unexpected, is a gift for which we ought to be deeply grateful, and also show our sense of God's mercy, by loving him more fervently and using all our members in His service.

And should not the rising again of our souls call still more for our gratitude? By the opening of the green leaves around us we are reminded of the Resurrection and the joyous future which lies before us all. Ice and snow and the cold winds of winter had bound nature as in a grave, but with spring the sun shines and pierces through the earth, giving light and heat and life to all things, while the grass and flowers spring up in splendid profusion. We have a beautiful figure here of what takes place in our souls. We have been lying slumbering in carelessness, apparently dead to all holy thoughts or wishes, when in love and pity our Sun of Righteousness shines upon us. What a change takes place! Suddenly we feel that there is new life in us, the life which is hid with Christ in God. What matters it to us then if our mortal bodies pass away for the time? Should it please our Heavenly Father to restore them to health, well, any way they are His which he made for His good pleasure, but they contain souls which His blessed Son has bought again with the price of His most precious Blood.

Just lately all Christendom has been turning its eyes upon the fearful spectacle of our Redeemer's Cross and Passion; now it is rejoicing in His triumph over sin and death. We know now that our Redeemer liveth, that by His rising again as on Easter Day, "Death is swallowed up in victory," and though our bodies may have crumbled as dust to dust, yet, at the last Day, we shall in our flesh see God.

### The Muses' Looking Glass.

### GOD'S FOOL.*

A book of no ordinary interest. The psychological problem which is its main motive is worked out with a rare combination of accurate deduction and imaginative power. The common stock-in-trade of love and marriage is abandoned, but vivid pictures of Dutch society serve to throw into relief the hero of the drama on whom, from first to last, the interest centres. Some idea of this unusual conception we propose to lay before our readers, but the book should be read.

A strange hero this Elias Lossell, called apart by God to be His fool. And yet visit him at his little villa on the outskirts of Koopstadt, and you shall not think him so unlike other heroes. For he is young, not much over thirty, and richer than any merchant in Koopstadt. Kindly, gentle and generous too, as a hero should be, and, above all, handsome, with golden hair and beard, noble forehead, high and pure, and lofty stature. Eyes he has, too, of wonderful, fathomless depth of sorrow and peace, and a voice like the sound of a trumpet falling strangely on the silence—a silence you fear to break in presence of one whose ears are sealed for ever from your voice, whose eyes behold unhindered that light alone which lighteth every man that cometh into the world. Blind and deaf. Twenty years before, a flower-pot, flung in play by his little stepbrother, Hubert, had inflicted on his brain an injury which had severed first his ears from hearing, then his eyes from sight, and then gradually his mind from knowledge. He could not learn, this fool, or remember new facts. What he knew at the time of the accident remained with him. He could talk as a child can talk, and reason as a child. So, too, he had become able to understand what was spelled out to him on his neck or hand, chiefly by his faithful nurse Johanna. And by her efforts many simple occupations and interests became open to him, for though the channels of intellect were choked, the road to his heart lay open. Those whom he loved he never forgot, and he loved all who were kind to him, all who were in want; only he would acknowledge half sadly "Elias loves Elias best of all." All the wealth of the great firm of Volderdoes Zonen was his. Only a few shares had fallen to the acting partners, his half-brothers; Hubert in China, vowed life-long devotion to the brother he had injured; Hendrik, the real manager, in Koopsdadt. But now Hubert is at home again, and his English wife has learnt to spell out to Elias her imperfect Dutch, completing thus by her teaching the lessons of the faithful nurse. "From Mother Margaretha's lips he now first heard the story of the Lord Christ, the child Christ, the Christ on the cross. He could not fathom it clearly, he could not always remember it accurately, but instinctively he accepted it. 'We would see Jesus,' say they who transparently see everything but Him. The blind man said, 'I see Him,' and was at rest. He saw Him because of the darkness? So be it. So do men see the stars." Long ago Elias had burned to give away the money which meant to him so little because "God made the rich to help the poor." And now he passionately pleads for leave to deprive himself of all the treasures which make up the sum of his scanty joys. "I don't think Christ had any flowers of His own that He would have kept. And then I shall be exactly like Christ." Ah, Elias, fool in men's sight, very near that likeness shalt thou approach, yet by a steeper road than thou canst now foresee.

All this time we have said little of Hendrik Lossell the clever, pitiful, mean little cad, on whom fortune has thrown the direction of the house of Volderdoes Zonen. Strictly tied up on Elias, the bulk of the capital lies out of the reach of the principal partner. Beguiled by his wife's brother Alers, he involves the capital of the firm in one reckless speculation after another until Hubert's return home full of suspicious watchfulness places him in a position of desperation. It becomes necessary to procure the signature of Elias for further access to the funds of the firm, and for this purpose Hendrik hastens late at night, with notary and deed prepared, to his lonely villa, only to find that Hubert's cautions have prevailed, and that Elias is firm in his refusal. The notary departs with the unsigned deed, and the two brothers remain alone in the deserted house till Elias's quick instinct warns him of another presence, and his call of alarm is answered by a violent blow on the forehead. Returning to consciousness, after an interval of stupor, he strikes out in quick passion, again and again, at him he believes to be his assailant, till his victim falls helpless before him on the ground.

That night all Koopstadt learns that Hendrik Lossell has been murdered by his idiot brother, and Elias, persuaded of the same himself, lies crushed with the horror of an unforgivable sin. "He had murdered his brother as the Jews murdered Christ." Meantime Hubert is relentlessly tracking down Alers, who was seen at the villa after the notary's departure, and whose motive for the crime is apparent in Hendrik's retention of a forged deed. And still Elias in his isolation believes himself guilty until Johanna elicits accidentally his impression that he has slain his brother by a blow with his fist. Now Hendrik had been stabbed. And when this is explained to Elias there comes back to him the memory of Hubert's presence in the room during his half-stupor, and a resistless conviction that the fatal blow was struck by no other. Few will read untouched the closing scene where Elias owning to equal guilt in intention, reveals to Hubert his knowledge of the true murderer. For Hubert it is, Hubert the Fatalist, whose creed has been that "God is dead," who has struck down Hendrik at the moment when his dishonesty stood declared, and planned to fasten upon Alers a retribution due in justice if not in law. And in his proud attitude of self-justified vengeance Hubert is confronted by the simple ethics of the "fool" who had striven to be "exactly like Christ." "I shall not tell anything about your coming to anybody. Never! But I shall say: 'Gentlemen, it was I who killed my brother. I was angry with him for striking me. You must lock me up.' And you must live to be very, very sorry, Hubert. . . . It will all come right, only we must pray very much. We must pray very much, dear brother." And so he went to his judges. And Hubert, left alone, stared stupidly for one moment at the door. Then he sprang forward with a cry no one but himself could hear, "Elias!"

And God's fool has gained his heart's desire. For by that willing sacrifice of his own life his brother's soul is saved.

* "God's Fool." By Maarten Maartens. 3 vols. (Bentley and Son.)

### Appointments.

[It is requested that successful candidates will send a copy of their applications and testimonials, with date of election, to The Editor, The Lodge, Porchester Square, W.]

Dunedin Hospital, New Zealand.—Miss Isabella Fraser has been appointed Matron and Superintendent of Nurses at the Dunedin Hospital. Miss Fraser was trained at the Royal Infirmary, Edinburgh, and takes with her many cordial good wishes. We congratulate the New Zealand Hospital on securing her services.

Wellington and District Cottage Hospital.—Miss T. E. Borlase has been appointed Nurse-Matron of the Cottage Hospital, Wellington, Somerset. Miss Borlase was trained at the General Infirmary, Leeds, and subsequently held appointments at the Metropolitan Asylums Board Western Hospital, and Gore Farm Hospital, also at Hounslow Cottage Hospital. Miss Borlase's varied experience specially fits her for her present appointment, and we wish her every success.

Llandudno Convalescent Home for Women.—Miss Mary Gardner has been made Matron of the Home for Women at Llandudno. Miss Gardner was trained and worked for three years in connection with the Mildmay Institute, London, and was afterwards Staff Nurse at the General Hospital, Birmingham. Since then Miss Gardner held the posts of Night Superintendent at the Cardiff Infirmary and at the Royal Hants Infirmary, Southampton, and she has recently taken temporary duty at the Queen's Hospital, Birmingham.

### Wants and Workers.

Nurse Lucie will be glad if any reader of The Hospital can give her address and any particulars of the Flower Mission.

### The Book World for Women and Nurses.

[We invite Correspondence, Criticism, Enquiries, and Notes on Books likely to interest Women and Nurses. Address, Editor, The Hospital (Nurses' Book World), 428, Strand, W.C.]

### TO OUR READERS.

With the view of meeting the wishes of a large circle of readers, we have determined to devote a special section of the "Nursing Mirror Supplement" to books and literary matters especially calculated to interest women and nurses. We are aware that, to very many, a careful classification of the book advertisements proves most helpful, and we shall endeavour to so arrange as to enable every reader of The Hospital to have information concerning every new book found of value. We shall be glad to give insertion to inquiries concerning rare or out-of-the-way books which may have a special interest of their own. We shall also welcome the co-operation of our readers to the extent of directing our attention to any British, Foreign, Colonial, or American books calculated to prove of service to women and nurses. Publishers will please direct all books intended for review to the Editor, 428, Strand, W.C.

### AN AMERICAN MAGAZINE.

Godey's Magazine (New York, 21, Park Row) for March contains a story of some length entitled "The Romance of a Trained Nurse," by Kate Upson Clark. Those members of the nursing profession who have found in the routine of hospital life little of the romance which gilded the picture of their imagination, will find in reading these pages that a very large amount of exciting variety can fall to the lot of the nurse in fiction. The nurse heroine of the tale has, like many other young women, long cherished a desire to "do" something, and has foundthe daily round of gaiety which constitutes her life unsatisfying. Her father on his dying bed explains to her his uneasiness that he is leaving her badly provided for, and practically dependent on her step-mother. Priscilla, our heroine, consoles him by confiding her desire to work, and receives his consent to her plan to become a nurse. When later on she informs her step-mother of her intention, she meets with some opposition on the score of loss of position. On this lady being assured that times have changed, and that to be a nurse is "quite the thing" nowadays, she becomes more reconciled to her step daughter's choice, who takes up her abode at St. Mary's Hospital, where a few months' residence proves her fitted in every way for the path she has chosen. After the lapse of two years the hero of the tale, a Dr. Engel, comes on the scene. He performs an operation on an idiot child. In the words of the narrative, "He had accordingly made an incision in the skull along the median line, extending from the frontal suture to the occipital suture, and removed part of the substance of the skull on the left side. The operation in the original case took place on May 9th, and from June 15th the child began to walk, to play with her doll, and, in short, to behave like other children." This descriptive little passage is fortunately the only attempt at medical detail. During this operation Priscilla shows herself highly capable. Dr. Engel requiring a nurse for the hospital over which he presides, she is chosen for the post, and placed in a responsible position. The description of the

### THE BOOK WORLD FOR WOMEN AND NURSES—*continued.*

arrangements in St. Jerome's Hospital will be interesting to English readers, as it reveals differences to our own hospital arrangements in a good many particulars. The fact that this particular hospital is a charity is especially dwelt upon, which brings out the difference of British and foreign systems of medical relief. Glimpses given us of the wards by means of the pictures (for the story is profusely illustrated) show them to be very much the same as our own. In the new hospital Priscilla's life becomes fraught with events. She is seriously injured by a patient she is endeavouring to assist; she saves a doctor's life from a burglar; the husband of a patient who dies in the hospital falls in love with her, and a fellow worker picks her out as an object for malicious attacks. We have carefully avoided touching on the main romance in her history, as there may be many who will like to peruse Priscilla's history for themselves, and we would not rob them of the interest of the dénounment beforehand. The tale, which is pleasantly written, is so charmingly illustrated that if for this reason alone one shilling will not be badly expended to obtain it.

### HOME NURSING.

Messrs. W. and R. Chambers have published a very useful and practical volume on Home Nursing, by Rachel A. Newmann. The directions for the various preparations—poultices, fomentations, and the like that are usually left in a nurse's hands, are clear and precise. The advice as to rest and exercise, through neglect of which so many amateur attendants on the sick destroy their usefulness within a few days, is thoroughly sensible, and the tone of the book is all that could be desired. It is kindly but self-controlled, as a true nurse should be, frankly stating the dangers and complications of each ailment dealt with, but neither alarmist nor emotional. We should be glad to see some such book as this introduced as a supplement to domestic economy into our national schools, for it is among the very class of our population where accidents and epidemics of childish diseases are most common that ignorance, lack of resource, and the hysterical distress that arise from these in face of danger are most frequently to be met with.

### Books Received.

Bailliere, Tindall, and Cox.

"The Dental Profession." By Henry Sewell.

"Practical Pharmacy for Medical Students." By A. Campbell Stark.

The Century Company, New York.

"A Handbook of Invalid Cooking." By Mary A. Boland.

**Periodicals, etc.**—"Light." "The Service of the King." "Medical Pioneer." "The Charity Organisation Review." "The Dietary of Troops." By Lieut. Colonel Wintle, R.A. "Humanity and Vegetarianism." "London." "The Therapist." "The Religious Review of Reviews." Religious Tract Society: "The Boy's Own Paper." "Girl's Own Paper." "Our Little Dots." "Hannah More." "Friendly Greetings." "Light in the Home." "Child's Companion." "The Cottager and Artisan." "The Young Gentlewoman." "Proceedings of the Society for the Study of Inebriety."

A Manual of Medical Treatment or Clinical Therapeutics, by Dr. J. Burney Yeo., F.R.C.P., will shortly be issued in two volumes, by Messrs. Cassell and Company, with numerous illustrations.

## Figures and Tables

**Figure f1:**